# First Report of *Alphacoronavirus* Circulating in Cavernicolous Bats from Portugal

**DOI:** 10.3390/v15071521

**Published:** 2023-07-08

**Authors:** Mahima Hemnani, Priscilla Gomes da Silva, Gertrude Thompson, Patricia Poeta, Hugo Rebelo, João R. Mesquita

**Affiliations:** 1 School of Medicine and Biomedical Sciences, Porto University, 4050-313 Porto, Portugal; up202110040@edu.icbas.up.pt (M.H.); up202002072@edu.icbas.up.pt (P.G.d.S.); gathompson@icbas.up.pt (G.T.); 2 Epidemiology Research Unit (EPIunit), Institute of Public Health, University of Porto, 4099-002 Porto, Portugal; 3 Laboratório Para a Investigação Integrativa e Translacional em Saúde Populacional (ITR), 4050-313 Porto, Portugal; 4 LEPABE—Laboratory for Process Engineering, Environment, Biotechnotlogy and Energy, Faculty of Engineering, University of Porto, 4099-002 Porto, Portugal; 5 ALiCE—Associate Laboratory in Chemical Engineering, Faculty of Engineering, University of Porto, 4099-002 Porto, Portugal; 6 Centro de Investigação em Biodiversidade e Recursos Genéticos, InBIO Laboratório Associado, Universidade do Porto, 4485-661 Vairão, Portugal; hugo.rebelo@cibio.up.pt; 7 Microbiology and Antibiotic Resistance Team (MicroART), Department of Veterinary Sciences, University of Trás-os Montes e Alto Douro, 5000-801 Vila Real, Portugal; ppoeta@utad.pt; 8 Associated Laboratory for Green Chemistry (LAQV-REQUIMTE), University NOVA of Lisbon, 1099-085 Caparica, Portugal; 9 Veterinary and Animal Research Centre (CECAV), University of Trás-os-Montes e Alto Douro, 5000-801 Vila Real, Portugal; 10 Veterinary and Animal Research Centre, Associate Laboratory for Animal and Veterinary Science (AL4AnimalS), 5000-801 Vila Real, Portugal; 11 ESS, Instituto Politécnico de Setúbal, 2910-761 Setúbal, Portugal

**Keywords:** coronavirus, cavernicolous bats, *Alphacoronavirus*, Portugal

## Abstract

The emergence of novel coronaviruses (CoVs) has emphasized the need to understand their diversity and distribution in animal populations. Bats have been identified as crucial reservoirs for CoVs, and they are found in various bat species worldwide. In this study, we investigated the presence of CoVs of four cavernicolous bats in six locations in the centre and south of Portugal. We collected faeces, anal, and buccal swab samples, as well as air samples from the locations using a Coriolis air sampler. Our results indicate that CoVs were more readily detected in faecal samples compared to anal and buccal swab samples. No CoVs were detected in the air samples. Phylogenetic analysis showed that the detected viruses belong to the *Alphacoronavirus* genus. This study represents the first report of Alphacoronaviruses circulating in bats in Portugal and highlights the importance of continuous surveillance for novel CoVs in bat populations globally. Ongoing surveillance for CoVs in bat populations is essential as they are a vital source of these viruses. It is crucial to understand the ecological relationships between animals, humans, and the environment to prevent and control the emergence and transmission of infectious diseases. Further ecological studies are needed to investigate the factors contributing to the emergence and transmission of zoonotic viruses.

## 1. Introduction

Coronaviruses (CoVs) are viruses belonging to the order *Nidovirales*, family *Coronaviridae*, and subfamily *Orthocoronavirinae*. They are positive-sense RNA viruses, with one of the largest genomes within RNA viruses [1]. They have an envelope with structures protruding from the surface called “spikes” [2]. CoVs have diverse animal hosts ranging from mammals to bird species, mainly causing enteric and respiratory diseases of varying severity [3] and are classified into four genera [4]. Alpha (α) and Beta (β) CoVs, which commonly cause disease in mammals, are considered pathogenic viruses. On the other hand, Gamma (γ) and Delta (δ) CoVs, also known as avian CoVs [5], evolved from CoVs originating from birds, mostly causing disease in avian species [6,7,8]. Due to their long genomes of around 30 kb, high recombination frequency and high mutation rates [9,10], CoVs have the potential to adapt to new host species with altered pathogenicity, without sacrificing important elements to continue viable, causing a broad spectrum of diseases [11].

Furthermore, the most iconic examples of viral spillover to humans occurred in 2002/2003, when a highly pathogenic human CoV causing severe acute respiratory syndrome emerged in China (SARS-CoV), causing outbreaks worldwide [12], and in 2012, when another CoV emerged in the Arabian Peninsula, the Middle East Respiratory Syndrome (MERS-CoV), also causing severe acute respiratory syndrome [13]. At the end of 2019, another CoV named SARS-CoV-2 emerged in the city of Wuhan, in China and has since then been the causative agent of the COVID-19 pandemic [14].

Many studies have repeatedly pointed to bats as the natural and primary reservoirs of various viruses that are closely related to other mammalian coronaviruses (CoVs), shedding insight on the critical role that bats play in CoV transmission and evolution and also highlighting these animals as a significant source of viral diversity and potential spillover events to other mammals including humans [11]. Bats are found to be hosts of at least 30 different CoVs with complete genome sequences available, and many more considering those without whole-genome sequences available [14]. In this way, they are considered mammals hosting the highest number of CoVs [1] and the evolutionary source for several human CoVs [15].

Regardless, studies have shown that bats have special traits that allow them to replicate and excrete viruses that are lethal to other mammals without displaying severe clinical indications of disease [4]. They have genetic changes in their immune system, which can protect them from the toxic development of infectious pathologies or prevent the manifestation of clinical signs after infection [1]. Moreover, they can display a decrease in body temperature [16], which is a strategy for reduced viral replication and pathogenesis [17], and their ability to coexist with pathogens [18]. Understanding how bats maintain a virus within a population is important for predicting spillover transmission events [4].

Additionally, other ecological characteristics may facilitate viral spread: bats have commensal relationships with viruses, and the bat virome is even associated with enhanced immunity [19]. Social organization in bats also contribute to the maintenance of the virus in the population [14]. Several species of bats form large colonies with many individuals, thus facilitating the spread of viruses in bat populations [20].

Characterizing the transmission of pathogens from wildlife to humans is an ongoing and critical scientific challenge. However, this endeavour is often impeded by various limitations, particularly in detecting and studying elusive wild species [21]. Understanding the dynamics of pathogen spillover events and their implications for public health requires overcoming these obstacles to gain a comprehensive understanding of zoonotic diseases [22]. This way, we can better mitigate the risks, improve early detection, and implement effective strategies for preventing and managing potential outbreaks.

Manual handling of bats to collect samples has some technical difficulties, since some roosts are physically inaccessible, and others are toxic or unsafe for humans to explore [23]. Moreover, studies that involve manually capturing these bats or accessing roosts are also disturbing for the bats [23] and they might end up changing roots because of this disturbance, which would be costly for the colony [24]. In one study, a decrease in bat population density was attributed to drastically reduced adult female survival rates, which was a direct result of human disturbance to the bat colony. This low survival or permanent emigration of adult females may be the primary reason for the decline of certain colonies experiencing disturbances, and it can have a significant impact on colony persistence [25].

Additionally, procedures that require accessing caves can be particularly harmful to bats that are hibernating because they can awake them and use up their fat reserves unnecessarily [26]. To overcome these difficulties, a non-invasive sampling technique that does not need direct contact with the bats could be used. Considering that SARS-CoV and MERS-CoV have been reported to be detected in air samples [27], and all the evidence supporting the airborne transmission of SARS-CoV-2 as one of the main drivers of the COVID-19 pandemic [28], studying bat CoVs presence in air might be an alternative non-invasive sampling technique to study CoVs among bat populations, as the viruses carried by them might be present in the air of these animals habitats.

In Europe, to date, there are 25 studies that have evaluated the presence of CoVs in bats: six studies in Italy, four in Germany, two in Holland, two in Ukraine, and one in Belgium, Bulgaria, Denmark, Finland, France, Hungary, Luxembourg, Romania, Slovenia, Spain, Sweden, Poland, and the United Kingdom. So far, there are no studies on the presence of CoVs in bats and in their environment in Portugal. Hence, the primary objective of this study is to investigate the presence and genetic features of CoVs in various types of bat cavernicolous roosts across Portugal. The study aims to comprehensively examine the occurrence and diversity of CoVs in different bat habitats, ranging from natural cave systems to large buildings.

To achieve this, prospective sampling and testing were conducted, targeting a diverse array of bat roosts distributed throughout the country. Moreover, considering the potential for airborne transmission of known coronaviruses such as SARS-CoV, MERS-CoV, and SARS-CoV-2, we also performed air sampling in closed habitat environments to assess the potential presence of bat CoVs in the air, as this could pose an alternative non-invasive method for monitoring bat CoVs that does not involve capturing of the animal to collect clinical samples. This information will contribute to the broader understanding of CoV diversity, their circulation patterns, and the potential for spillover events. The findings of this study can also have significant implications for both bat conservation and public health.

## 2. Materials and Methods

**Sampling location**

**Figure 1:**
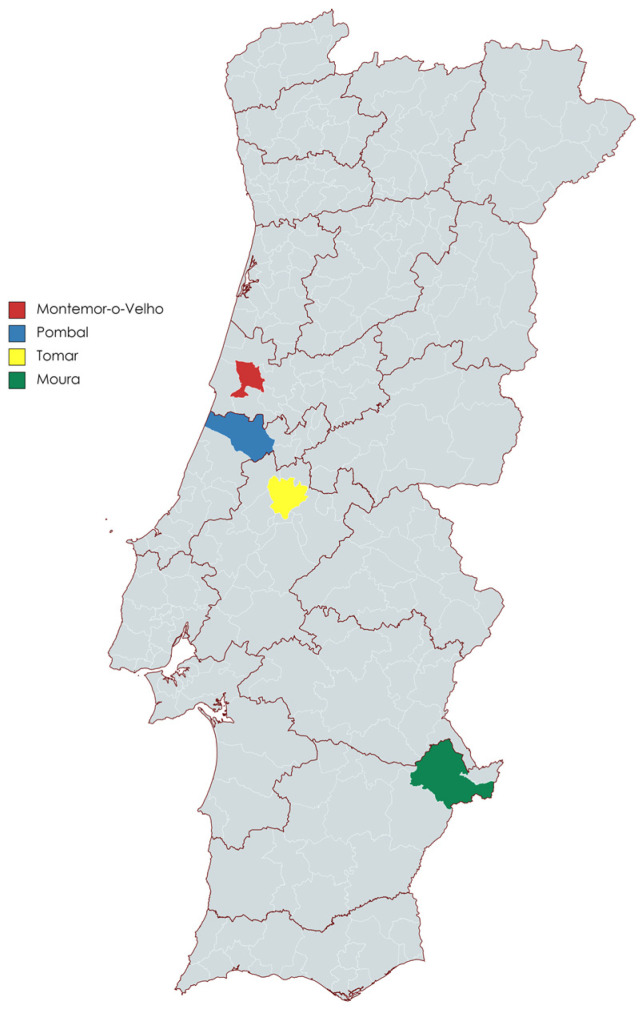
Selected sampling locations in Portugal used in this study.

Air and bat sampling was carried out during July 2022, at six locations in the centre and south of Portugal, namely two large historical buildings and four caves in the municipalities of Montemor-o-Velho, Pombal, Tomar, and Moura (Figure 1).

Air sampling was performed in each of these locations using a Coriolis Compact^®^ air sampler. The sampler was placed in the middle of each cave, at approximately 1.3 m in height. Each sampling was performed for 60 min with a 50 L/min airflow rate. After sampling, 4 mL of PBS was added to the sampling cones and the samples were immediately stored at 4 °C for transport to the laboratory until further processing.

Bats were captured using hand nets to collect specimens for study. We captured a total of 42 bats, belonging to three different genera and four different species: *Myotis myotis*, *Miniopterus schreibersii*, *Rhinolophus mehelyi*, and *Rhinolophus ferrumequinum*. The captured bats were handled carefully to ensure their well-being throughout the process and morphological identification was conducted by expert Hugo Rebelo.

During the handling procedure, anal swabs were taken from each bat, resulting in a total of 42 anal swab samples. Additionally, buccal swabs were also collected from all 42 bats. Furthermore, whenever faeces were shed during the collection procedure, they were collected as well, resulting in a total of 14 stool samples. Overall, we obtained a comprehensive set of 98 samples, consisting of anal swabs, buccal swabs, and stool samples from the captured bats. Details of the samples collected can be found in Table 1.

After the collection, the bats were promptly released back into their natural habitat. This is important to minimize any disturbance to their normal behaviour and preserve the integrity of the study population. All procedures related to bat capture and handling were carried out in strict compliance with the permits issued by the Instituto da Conservação da Natureza e Florestas, ensuring adherence to the regulations and guidelines set forth by the conservation authority.

**Screening for coronaviruses**

The samples were stored at −20° until further processing. Anal and buccal swabs were homogenized by vortexing in 500 µL of PBS pH 7.2. RNA was extracted from the faecal suspension using the QIAamp viral mini kit (Qiagen, Hilden, Germany) according to the manufacturer’s instructions using 140 µL of the cleared supernatants (after 1400× *g* for 2 min). The eluted RNA was then kept at −80 °C until further processing.

The extracted RNA was tested for CoVs using a broad-spectrum pan-CoV nested RT-PCR assay targeting the conserved region of RNA-dependent RNA polymerase (RdRp) with a final product size of 440 bp [10]. The sensitivity of the nested pan-CoV primers has been evaluated by comparing them with various protocols. This evaluation involved combining primers from different studies to achieve optimal performance. The aim was to enhance the chances of detecting both known and unknown coronaviruses from diverse sample sources [10]. It has been reported that utilizing a small partial region of the RdRp (RNA-dependent RNA polymerase) of coronaviruses is adequate for determining subgenus-level taxonomic classifications. This classification accuracy is comparable to that achieved using complete genome sequences [29]. For the first round of PCR, we used the One-Step RT-PCR kit (GRiSP^®^, Porto, Portugal). Amplification reactions with positive and negative controls were performed in Veriti 96 Well Thermal with the following conditions: initial cycle of 3 min at 95 °C (enzymatic activation, denaturation of the DNA template), followed by 40 cycles at 95 °C for 15 s, 50 °C for 15 s, and 72 °C for 2 s, with a final elongation at 72 °C for 10 min. For the second run, 2 µL of the first run products were used as templates in the Xpert Fast Hotstart Mastermix (2×) with dye (GriSP^®^, Porto, Portugal). PCR was performed in a final volume of 25 µL. The amplification reactions with positive and negative controls were carried out in the same thermocycler with the following conditions: the initial cycle of 3 min at 95 °C (enzymatic activation, denaturation of the DNA template), followed by 40 cycles at 95 °C for 15 s, 52 °C for 15 s and 72 °C for 2 s, with a final elongation at 72 °C for 10 min.

PCR amplification products were subjected to electrophoresis at 120 V for 30 min on a 1% agarose gel stained with Xpert Green Safe DNA gel stain (Grisp, Porto, Portugal) and then irradiated with UV light to identify target DNA fragments. A DNA weight comparison was used for measurements (100 bp DNA ladder; Grisp, Porto, Portugal).

**Sanger sequencing and phylogenetic analysis**

Positive amplicons were then purified with the GRS PCR Purification Kit (Grisp, Porto, Portugal) and, using Sanger sequencing, bidirectional sequencing was performed with the specific primers of the target gene. The sequences were then aligned with the software package BioEdit Sequence Alignment Editor v7.1.9, version 2.1 (Ibis Biosciences, Carlsbad, CA, USA) and compared with the sequences available in the NCBI nucleotide database (GenBank, Carlsbad, CA, USA) (http://blast.ncbi.nlm.nih.gov/Blast, accessed on 13 February 2023). The sequences obtained were included for phylogenetic analysis and submitted to GenBank under the accession numbers (OQ613363–OQ613369).

These sequences, together with 41 reference strains from the 4 CoV genera (Alpha-, Beta-, Gamma-, and Deltacoronavirus) obtained from GenBank, were aligned using MEGA 11 software [30]. Models function on MEGA 11 was used to opt for the model with the smallest Bayesian information criterion (BIC) score [31] using the maximum likelihood method, based on the general time reversible model using a discrete Gamma distribution and assuming evolutionarily invariable sites, 1000 bootstraps replicated, followed by editing with the Interactive Tree of Life (iTOL) platform [32].

## 3. Results

In the study, a total of six air samples were collected and none tested positive for CoVs. However, out of the 98 samples obtained from bats, seven samples (8.87%) exhibited amplicons of the expected size. These seven samples were further analysed through bidirectional sequencing and nucleotide BLAST analysis. The results revealed that all seven samples were characterized as *Alphacoronavirus*.

Interestingly, although a smaller number of stool samples were analysed compared to anal and buccal swabs, the stool matrix yielded the highest number of positive results with six samples testing positive. In contrast, only one anal swab and no buccal swabs showed positive results. It is worth noting that the anal swab that exhibited a positive result also corresponded to a positive stool sample, both obtained from a *Miniopterus schreibersii* (AN25 and F25).

Overall, the identified CoVs were found in different bat species. Two samples were from *Myotis myotis*, three from *Miniopterus schreibersii*, one from *Rhinolophus mehelyi*, and one from *Rhinolophus ferrumequinum*. For further information and specific sample details, refer to Table 2 of the study.

**Figure 2:**
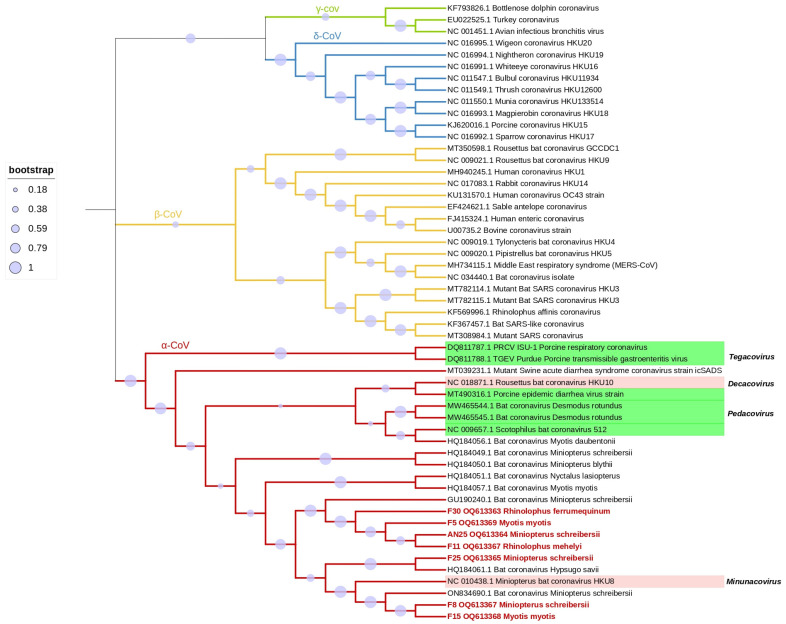
Phylogenetic tree constructed for the alpha, beta, gamma, deltacoronavirus and the alphacoronaviruses subgenus indicated in green and pink, using 46 reference strains and 7 strains identified in this study. Phylogenetic analysis was based on a 406 nt partial region of the RdRp. The tree was constructed using MEGA 11 and using the maximum likelihood based on the GTR + G + I model, and 1000 bootstraps were replicated. Samples from this study are indicated in red with the description of sample number, GenBank accession number and host bat species.

Sequence analysis conducted on the acquired CoV sequences revealed significant similarities to sequences obtained from bats discovered in Bulgaria, Italy, and Spain. The identities ranged from 93% to 100%, indicating a close relationship between the CoV strains circulating in European bats. Further characterization through BLAST analysis indicated that the sequences exhibited the strongest matches with CoVs identified in *Miniopterus schreibersii* (n = 6) and *Hypsugo savii* (n = 1) from Bulgaria/Italy and Spain, respectively. To confirm the classification, a phylogenetic analysis was performed using the seven obtained CoV sequences along with 41 reference strains. The analysis affirmed their placement within the *Alphacoronavirus* genus, as depicted in Figure 2.

## 4. Discussion

In this study we aimed to investigate the circulation of CoVs in two distinct epidemiological aspects: airborne CoVs at bat roosts and CoVs found specifically in cavernicolous bats in Portugal. This study represents the first-ever description of CoVs in bats in the country, providing crucial insights into the viral ecology and diversity of these animals. In total, 42 individuals were screened for CoVs by nested RT-PCR followed by sequencing. In this study, CoVs were not detected in the air. However, the detection was primarily observed in faeces samples (n = 6), suggesting that virus replication occurs in the gastrointestinal tract, highlighting the potential for fecal-oral transmission routes. The CoV strains found in the bat populations in our study are closely related to *Alphacoronavirus* strains retrieved from the bat species *M. schreibersii* from Bulgaria and Italy and *Hypsugo savii* from Spain. In the phylogenetic tree based on partial RdRp gene, the sequences in our study clustered with other members of the genus *Alphacoronavirus*, supported by 90% bootstrap value. Our sequences clustered with the same reference strains as indicated in Table 2.

The bat species sampled in this study do not migrate over long distances [33], hence no long distance transmission has likely occurred and viruses are probably circulating solely in the studied region. The identified bat CoVs clustered together but not according to the species. The sequences exhibited the strongest matches with CoVs identified in *Miniopterus schreibersii* and *Hypsugo savii* bat species, from Bulgaria/Italy and Spain, respectively, which suggests a potential lack of association between the bat species and the CoV strains under investigation. These findings point towards a broad CoV host range within the Chiroptera order, but further studies characterizing the CoVs full length genomes are necessary in order to make more definitive conclusions. As such, these viruses seem to not evolve within a certain bat species, but instead geographical location appears to have had a greater influence on the evolution and spread.

Our approach for the detection and characterization of CoVs has resourced to a partial RdRp region with primers described by [10] because according to the authors, this region was sufficiently informative to allow classification within known CoV genera. The RdRp exhibits a certain degree of sequence conservation across different CoVs subgenus. Focusing on this specific region, we can obtain valuable taxonomic information without the need for analyzing the entire viral genome and is highly effective in determining taxonomic classifications, reaching the subgenus-level [29].

To date, both Alpha and Beta CoVs have been found in bats [14]. Bat CoVs are known to be excreted at higher viral loads in stools, making the enteric route a major environmental source for the CoV spillover events [34]. The CoVs detected in this present study were detected mostly in feces, confirming the enteric route of transmission as the most significant, being consistent with other studies with bats [35,36]. The fecal-oral route has also been described in CoVs from other animals such as with feline coronavirus (FCoV), canine coronavirus (CoV) and swine coronavirus (SADS-CoV)—all of them classified as Alpha-CoV. The replication of CoVs in the enteric location suggests an adaptation of the virus to the bat host’s gastrointestinal environment. Further investigations into the mechanisms underlying this viral replication in the gastrointestinal tract may help unravel the unique interplay between the CoVs and the bats’ immune system. However, it is worth noting that the detection of CoVs exclusively in feces samples in this study does not completely rule out the possibility of other modes of transmission, such as respiratory or direct contact routes.

Previously, it has been reported the presence of SARS-CoV [37,38] and MERS-CoV [39,40,41] in air samples, with the discussion of the airborne route of transmission for these human CoVs gaining notoriety during the SARS-CoV-2 pandemic, with many reports of SARS-CoV-2 presence in indoor and outdoor samples throughout the world [27,42,43,44] and the World Health Organization acknowledging the airborne route as a transmission route for SARS-CoV-2 [45]. In response to the aforementioned findings, we conducted air sampling within bat caves and buildings to investigate the potential presence of CoVs in the air within these environments. Despite our efforts, we were unable to detect any CoVs from the collected air samples. Several factors could have contributed to this lack of detection, and we hypothesize that sampling conditions played a significant role. One potential factor that might have influenced our results is the sampling duration. The duration of air sampling plays a crucial role in capturing an adequate number of airborne particles including viral particles. If the sampling duration was insufficient, it could have led to a lower likelihood of capturing CoVs present in the air. Little is known on the airborne route of bat CoVs and the possibility of low viral copy excretion, generating aerosols with undetectable loads, could be also the case in bats. Therefore, it is possible that the duration of our air sampling was not optimized for the detection of CoVs, resulting in negative findings.

Additionally, the type of sampler used in this study was cyclone-based. While cyclone-based samplers are commonly employed for air sampling, they might not be the most effective option for capturing CoVs. Notwithstanding, both the choice of air sampler and duration of air sampling has been successfully applied in detecting SARS-CoV-2, an airborne CoV [27,46,47]. All in all, it is important to acknowledge these limitations and consider alternative sampling strategies for future investigations such as alternative sampling durations and utilizing samplers specifically designed for capturing viral particles that could enhance the sensitivity of CoV detection in the air [48].

When analysing these results it is also important to emphasize that virus concentrations in air can be low at the place and time of collection, which might yield negative results that do not necessarily mean that there was no virus present in the air at the moment of collection [46]. Further attempts of air sampling in bats environments should be performed with adjustments to the protocol such as longer sampling times and theuse of different air samplers that might be more suitable for sampling airborne pathogens such as samplers using the impinger, filter, and water-based condensation methods. Also, our air sampling was conducted during a dry summer and there may be other times of the year that could host more favourable climatic conditions for the virus persistence in the air.

Prior to this study, nothing was known about the diversity of CoVs in bats or in the air where bats are found in mainland Portugal where 27 species are acknowledged to occur, and now our results show that there are *Alphacoronaviruses* circulating in cavernicolous bats from Portugal, and they are closely related to bat species from Bulgaria, Italy, and Spain. Anthropogenic changes such as deforestation, habitat fragmentation, land-use, agriculture, and urbanization can promote the transmission of infectious diseases by increase the chances of human contact with bats [14]. Since bats are likely to harbour more than one species of viruses at the same time, which might allow the CoVs to incorporate genes from other viral families through recombination events [49], it is important to continue monitoring CoVs in bats and draw the baseline for future surveillance [35] where faecal sampling can play a relevant role, as demonstrated in this study. This may help to better understand the evolution and ecology of this group of viruses, their epidemiology, and the transmission of viruses between bats and between bats and humans and other animals.

## 5. Conclusions

This manuscript presents significant insights into the prevalence and genetic diversity of coronaviruses (CoVs) in bats and the air of bat roosts in Portugal. These findings are valuable for comprehending the epidemiology and ecology of CoVs, which are critical for effective public health interventions in controlling viral spillover events and spread. Our study identified CoVs in faecal samples from bats, indicating that gastrointestinal transmission is a likely route. This aligns with prior research demonstrating that bat faeces play a pivotal role in the environmental shedding of CoVs. The detection of CoVs in multiple bat species suggests that the virus can circulate between different bat populations. It is important to acknowledge the limitations of the sampling conditions and techniques employed as the study did not detect CoVs in air samples from bat roosts. Further investigation is needed to explore the airborne transmission of CoVs and optimize sampling strategies to capture airborne viruses in bat habitats. The manuscript underscores bats’ role as natural reservoirs of CoVs, showcasing their ability to replicate and excrete viruses without displaying severe clinical symptoms. Bats possess unique genetic and physiological adaptations that enable them to coexist with pathogens, reducing viral replication and pathogenesis. Additionally, their social organization and large colony sizes facilitate viral spread within bat populations. These findings significantly contribute to the global understanding of CoV ecology and transmission dynamics. They highlight the importance of ongoing research on bat viromes and the surveillance of CoVs in bats and their environments. Detecting and characterizing transmission events from wildlife to humans remains a substantial scientific challenge, but it is vital for improving public health efforts and mitigating the risk of future viral spillover events. These findings underscore the need for continuous research and surveillance to mitigate the dangers associated with zoonotic diseases, thus expanding our knowledge of CoV ecology and transmission dynamics.

## Figures and Tables

**Table 1:** Details of the species found according to each location, number of individuals found in each location, matrices collected, and quantity of each matrices collected.

Location	Species	Number of Individuals Found in Each Location	Matrices Collected		
			Stool Samples	Anal Swab	Buccal Swab
Pombal	*Myotis myotis*	1	0	1	1
Tomar	*Miniopterus schreibersii*	7	1	7	7
	*Rhinolophus mehelyi*	2	1	2	2
	*Myotis myotis*	5	3	5	5
Moura	*Miniopterus schreibersii*	7	2	7	7
	*Rhinolophus mehelyi*	6	1	6	6
	*Myotis myotis*	9	4	9	9
	*Rhinolophus ferrumequinum*	5	2	5	5
Total		42	14	42	42

**Table 2:** Details of the positive samples from this study, where F represents feces samples, and AN represents anal swabs.

Collection Site	Sample ID	Host Species	Acession Number	Shared Identity
Tomar	F5	*Myotis myotis*	OQ613369	*Miniopterus schreibersii*-Bulgaria-GU190240.1
	F8	*Miniopterus schreibersii*	OQ613367	*Miniopterus schreibersii*-Italy-ON834690.1
	F11	*Rhinolophus mehelyi*	OQ613367	*Miniopterus schreibersii*-Bulgaria-GU190240.1
	F15	*Myotis. Myotis*	OQ613368	*Miniopterus schreibersii*-Bulgaria-GU190240.1
Moura	F25	*Miniopterus schreibersii*	OQ613365	*Hypsugo savii*-Spain-HQ184061.1
	AN25	*Miniopterus schreibersii*	OQ613364	*Miniopterus schreibersii*-Bulgaria-GU190240.1
	F30	*Rhinolophus ferrumequinum*	OQ613363	*Miniopterus schreibersii*-Bulgaria-GU190240.1

## Data Availability

The data presented in this study are available on request from the corresponding author.
